# Dorsal Root Ganglia Volume—Normative Values, Correlation with Demographic Determinants and Reliability of Three Different Methods of Volumetry

**DOI:** 10.3390/diagnostics12071570

**Published:** 2022-06-28

**Authors:** Moritz Kronlage, Thomas David Fischer, Rouven Behnisch, Daniel Schwarz, Philipp Bäumer, Veronique Schwehr, Sabine Heiland, Martin Bendszus, Tim Godel

**Affiliations:** 1 Department of Neuroradiology, Heidelberg University Hospital, Im Neuenheimer Feld 400, 69120 Heidelberg, Germany; moritz.kronlage@med.uni-heidelberg.de (M.K.); thomas-david.fischer@gmx.de (T.D.F.); daniel.schwarz@med.uni-heidelberg.de (D.S.); p.baeumer@dialog-aoe.de (P.B.); veronique.schwehr@gmx.de (V.S.); sabine.heiland@med.uni-heidelberg.de (S.H.); martin.bendszus@med.uni-heidelberg.de (M.B.); 2 Institute of Neuroradiology, LMU Munich University Hospital, Marchioninistr. 15, 81377 Munich, Germany; 3 Institute of Medical Biometry and Informatics, Heidelberg University, Im Neuenheimer Feld 130.3, 69120 Heidelberg, Germany; behnisch@imbi.uni-heidelberg.de; 4 Center for Radiology dia.log, Vinzenz-von-Paul Str. 8, 84503 Altötting, Germany

**Keywords:** magnetic resonance neurography, dorsal root ganglia, demographic determinants, imaging marker

## Abstract

**Background:** Dorsal root ganglia (DRG) volume assessment by MR-Neurography (MRN) has evolved to an important imaging marker in the diagnostic workup of various peripheral neuropathies and pain syndromes. The aim of this study was (1) to assess normal values of DRG volume and correlations with demographic determinants and (2) to quantify the inter-reader and inter-method reliability of three different methods of DRG volumetry. **Methods:** Sixty healthy subjects (mean age: 59.1, range 23–79) were examined using a 3D T2-weighted MRN of the lumbosacral plexus at 3 Tesla. Normal values of DRG L3 to S2 were obtained after exact volumetry based on manual 3D segmentation and correlations with demographic variables were assessed. For the assessment of inter-reader and inter-method reliability, DRG volumes in a subset of 25 participants were measured by two independent readers, each applying (1) exact volumetry based on 3D segmentation, (2) axis-corrected, and (3) non-axis-corrected volume estimation. Intraclass correlation coefficients were reported and the Bland–Altman analysis was conducted. **Results:** Mean DRG volumes ranged from 124.8 mm^3^ for L3 to 323.3 mm^3^ for S1 and did not differ between right and left DRG. DRG volume (mean of L3 to S1) correlated with body height (r = 0.42; *p* = 0.0008) and weight (r = 0.34; *p* = 0.0087). DRG of men were larger than of women (*p* = 0.0002); however, no difference remained after correction for body height. Inter-reader reliability was high for all three methods but best for exact volumetry (ICC = 0.99). While axis-corrected estimation was not associated with a relevant bias, non-axis-corrected estimation systematically overestimated DRG volume by on average of 15.55 mm^3^ (reader 1) or 18.00 mm^3^ (reader 2) when compared with exact volumetry. **Conclusion:** The here presented normal values of lumbosacral DRG volume and the correlations with height and weight may be considered in future disease specific studies and possible clinical applications. Exact volumetry was most reliable and should be considered the gold standard. However, the reliability of axis-corrected and non-axis-corrected volume estimation was also high and might still be sufficient, depending on the degree of the required measurement accuracy.

## 1. Introduction

High-resolution imaging of the peripheral nervous system (PNS) by Magnetic Resonance Neurography (MRN) has become a well-established diagnostic tool in the localization of peripheral nerve lesions and in the investigation of pathophysiological processes in peripheral polyneuropathies (PNP) caused by compression, trauma, metabolic or inherited disorders [1,2,3]. Previous studies identified dorsal root ganglia (DRG) as a vulnerable site of peripheral nerve damage in various inherited and metabolic PNS disorders, e.g., in Fabry disease, Neurofibromatosis type 2 or in diabetic neuropathy [1,4,5,6,7,8], where pathological alterations are associated with changes in DRG volume and perfusion.

As a consequence of their close anatomical relationship to the central nervous system (CNS) interface, their internal anatomical-structural organization (central vs. a peripheral zone), and their unique blood supply, primarily sensory neurons of the DRG are considered a promising therapeutic target in peripheral neuropathies and pain conditions [9]. 

Despite novel functional MRN techniques that are capable of providing additional quantitative information, e.g., alterations in DRG perfusion, measurement of DRG volume and T2-signal represent the most feasible and meaningful MRN biomarker [3,8]. In order to establish DRG volume as a quantitative biomarker in clinical routine, it is essential to define valid normal values and reliable measurement protocols. Moreover, it is crucial to understand potential associations of DRG volume with demographic determinants in healthy subjects, as such associations have been reported for other morphological and functional MRN parameters such as nerve cross sectional area, fractional anisotropy or magnetization transfer ratio [10,11,12].

The DRG volume may be measured using different methods in a 3D MR neurography sequence [13,14,15]. Exact volumetry is based on 3D segmentation of the whole DRG and requires manual segmentation of the DRG contours in multiple slices. While this technique is expected to be the most precise, it is also the most time-consuming [16,17,18]. Alternatively, a simple estimation of DRG volume may be based on the measurement of three orthogonal diameters (A, B, and C) and the formula of the volume of an ellipsoid (V = π/6 × A × B × C) [16,17,18]. For axis-corrected measurement, these diameters are obtained in reformations, which are individually aligned with the long axis of each DRG. Non-axis-corrected estimation is less time-consuming, since all diameters are simply obtained in standard axial and coronal reformations, which do not exactly respect the exact orientation of the individual DRG. The future use of DRG volume as a clinical biomarker requires a systematic assessment of reliability of DRG volumetry [5,7,8,19]. While several studies have assessed DRG volume in specific peripheral neuropathies and control cohorts, data on reliability of DRG volumetry and quantification of the measurement error are still scarce [1,4,5,6,7,8]. During the preparation of this manuscript, one study was published, which provided evidence that estimation based on the ellipsoid formula may potentially underestimate true DRG volume [13]. 

The hypothesis of this study was that (1) DRG volume depends on demographic determinants and (2) DRG volume estimation by V = π/6 × A × B × C simplification is associated with a particular measurement error of largely unknown significance and extent. Thus, the approach of this study was twofold; (1) to assess normal values of DRG volume and potential correlations with demographic determinants in a cohort of 60 healthy participants and (2) to quantify both inter-reader and inter-method reliability for the three different methods of DRG volumetry in a subset of 25 subjects.

## 2. Patients and Methods

### 2.1. Clinical and Demographic Patient Data

This study was performed in accordance with the Declaration of Helsinki, approved by the institutional ethics board of the Medical Faculty of Heidelberg University (S-398/2012, version 6, 26 April 2018) and written informed consent was obtained from all participants. Overall, 60 healthy adolescents (30 males, 30 females, mean age 50.1 years, range 23–79 years, 5 men and 5 women in each decade) were recruited prospectively by public announcement and investigated by a standardized MRN protocol as previously described [11]. Inclusion criteria were age 20 to 80 years, absence of diabetes mellitus, neuropathic pain or other sources of acute or chronic pain (e.g., sudeck atrophy, fibromyalgia), and any other concomitant disease as risk factors for peripheral PNP (e.g., inherited or metabolic disorders with PNP as major a complication such as neurofibromatosis, amyloidosis, or hereditary neuralgic amyotrophy). Exclusion criteria were any contraindications for MRI and previous surgeries related to the PNS. Sex, age, body height and weight were documented as demographic determinants.

### 2.2. Imaging Protocol

Examinations were conducted on a 3 Tesla Magnetic Resonance scanner (Magnetom TRIO, Siemens Healthineers, Erlangen, Germany). A 15-channel transmit/receive spine coil and an 8-channel receive body flex coil (Siemens Healthcare) were used for imaging of the lumbosacral plexus. All 60 subjects underwent high-resolution MR-Neurography protocol including a 3D T2-weighted SPACE (Sampling-Perfection-with-Application-optimized-Contrasts-using-different-flip-angle-Evolution) STIR (Short-Tau-Inversion-Recovery) sequence of the lumbosacral plexus with the following parameters: repetition time/echo time 3000/208 ms, effective echo time 68 ms, inversion time 210 ms, field of view 305 × 305 mm^2^, matrix size 320 × 320 × 104, slice thickness 0.95 mm, voxel size 0.95 × 0.95 × 0.95 mm^3^ and acquisition time 8:35 min. 

### 2.3. Imaging Analysis

Image post-processing was performed by two independent raters with 5 and 8 years of experience in musculoskeletal imaging (D.F. and T.G.) who were blinded to each other and to demographic determinants. Assessment of DRG volume was performed using Digital Imaging and Communications in Medicine (DICOM) viewing software Osirix (Pixmeo, Bernex, Switzerland, version 12.5.2). 

**Figure 1:**
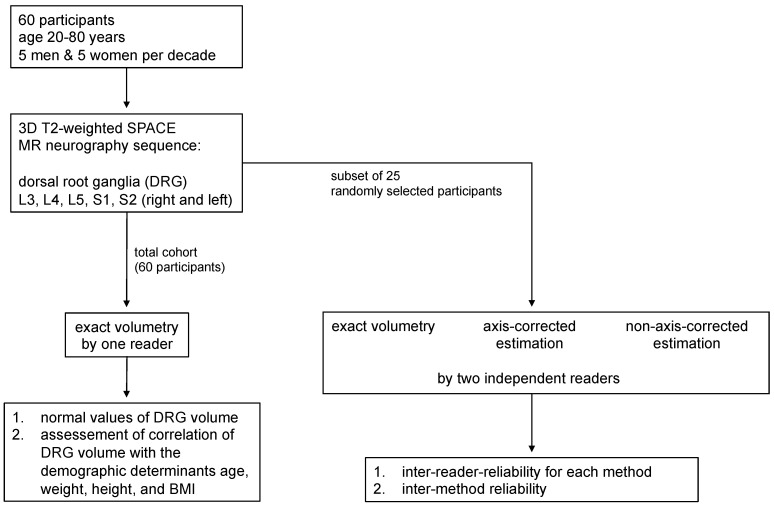
Flowchart of study design. 60 participants were examined with a 3D MR neurography protocol including the dorsal root ganglia (DRG) L3-S2. Normal values of DRG volume and correlation analyses with demographic determinants were based on assessment of the whole cohort by one reader using exact volumetry. In order to determine inter-reader reliability and inter-method reliability, DRG volumes were additionally obtained in a subset of 25 participants by two independent readers and according to three methods (exact volumetry, axis-corrected estimation and non-axis-corrected estimation). SPACE = Sampling-Perfection-with-Application-optimized-Contrasts-using-different-flip-angle-Evolution.

**Figure 2:**
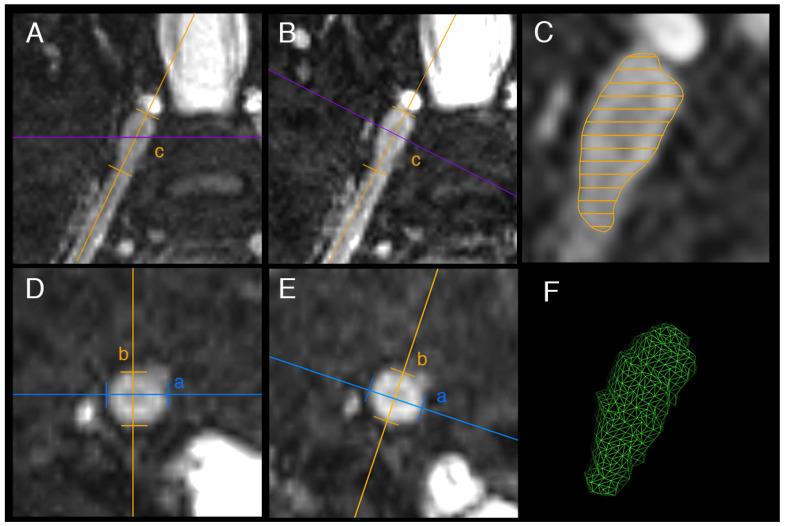
Exemplary images of dorsal root ganglion (DRG) volumetry. For non-axis corrected volume estimation the DRG main axes a, b, and c were measured in standard anatomical reformations ((**A**): coronal, (**D**): axial). For axis-corrected volume estimation, main axes were measured in coronal (**B**) and axial (**E**) reformations, that were aligned with the orientation of the individual DRG. Both methods of volume estimation rely on the same formula of an ellipsoid V = π/6 × a × b × c. For exact volumetry, the volume of the DRG was segmented in multiple axial planes ((**C**) showing coronal view with axial planes symbolized as parallel lines). Subsequently, a 3D model was created (**F**) and volume was calculated.

The study design is summarized in Figure 1. For the assessment of normal values of DRG volumes and correlation analyses with demographic determinants, DRG volumes L3 to L2 were assessed in all sixty participants by exact volumetry based on 3D segmentation. For this, the contour of each DRG circumference was defined by D.F. using slice-by-slice by manual segmentation in the axial and coronal reformation (Figure 2C,F). Subsequently, a 3D model was created, and volume calculation was performed. For correlation analyses with demographic determinants, one mean DRG volume per participant was calculated for the DRG L3 to L1 from both sides. The right DRG S2 was outside the field of view (FOV) in four and the left DRG S2 was outside the FOV in five participants. Therefore, S2 was not included in the mean DRG volume.

Inter-reader reliability and reliability between different measurement methods was assessed in a subset of 25 randomly selected participants (Figure 1). In these participants, DRG volumes L3 to L2 were assessed by (1) exact volumetry, (2) axis-corrected volume estimation, and (3) non-axis-corrected volume estimation by the two readers D.F. and T.G., independently. Exact volumetry was performed as described above. Axis-corrected and non-axis-corrected volume estimation were performed by using the formula for volume calculation of an ellipsoid (V = π/6 × A × B × C), whereas A represents the diameter in the axial plane, B the diameter at 90° in the axial plane, and C the length of the DRG’s long axis. For axis-corrected volume estimation, individual multiplanar reformations were acquired along and perpendicular to the long axis of each DRG, whereas for non-axis-corrected volume estimation, all DRG diameters were measured in the same axial and coronal reformations of the respective participants (Figure 2). As a supplementary analysis (Supplementary Figure S1), DRG volume calculation was additionally performed by the formula V = 2/3 × A × B × C + 75 mm^3^, which has recently been proposed by Weiner et al. for DRG volume calculation as an alternative to the ellipsoid formula [13].

### 2.4. Statistical Analysis

Statistical analyses and data visualization were performed with GraphPad Prism 9.0 (GraphPad Software, San Diego, CA, USA). *p*-values of ≤0.05 were considered significant. Paired *t*-tests were used to compare right- and left-sided DRG volumes for each segment with Bonferroni correction to correct for multiple testing. Pearson’s correlation coefficient r was reported for all correlation analyses of DRG volume with demographic variables. Unpaired *t*-test was used to compare DRG volumes between men and women. Interclass correlation coefficients (ICCs) were calculated according to Shrout and Fleiss [20]. For inter-reader reliability, a single measurement, absolute agreement, two-way random effects model ICC (2,1) was used. For inter-method reliability, a single measurement, absolute agreement, two-way mixed effects model was used. According to Koo and Li, ICC values between 0.5 and 0.75 were considered moderate, between 0.75 and 0.9 were considered good and greater 0.9 were considered excellent agreement [21]. To compare mean values of DRG volume for each measurement method, a one-way analysis of variance (ANOVA) with Geisser–Greenhouse correction and Tukey correction for multiple comparisons was calculated and the adjusted *p*-values were reported. All results are documented as mean values ± standard deviation. 

## 3. Results

### 3.1. Demographic Characteristics of Subjects

The study cohort consisted of 60 healthy subjects (30 males, 30 females), aged between 23 and 79 years, as described in a previous study investigating peripheral nerve caliber and T2 relaxometry in healthy volunteers [11]. Mean age was 50.08 ± 17.28 years, body height was 174.20 ± 9.59 cm and body weight was 75.42 ± 16.54 kg (Supplementary Table S1). Male and female participants did not differ in age (men: 50.47 ± 17.38 years; women: 49.70 ± 17.48 years, *p* = 0.87), but differences were found in height (men: 181.23 ± 6.10 cm; women: 167.17 ± 6.80 cm, *p* < 0.001) and weight (men: 86.77 ± 13.61 kg; women: 64.07 ± 10.23 kg, *p* < 0.001). 

### 3.2. Normal Values of DRG Volumes and Correlations with Demographic Variables

**Figure 3:**
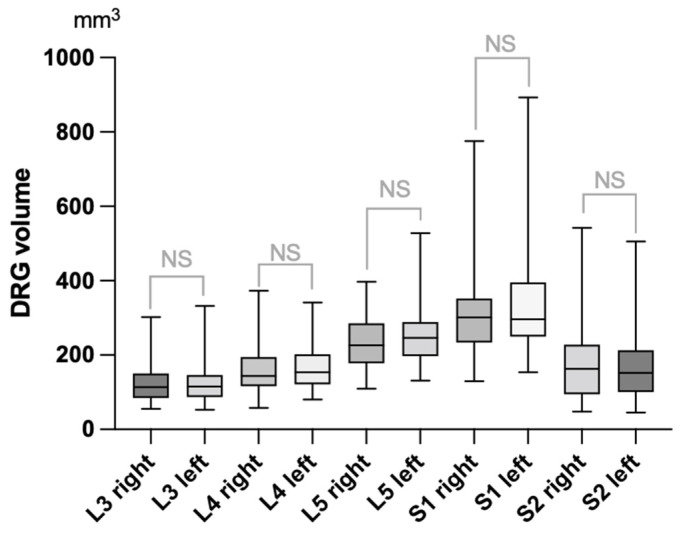
Box-and-whisker plot of dorsal root ganglia (DRG) volumes as assessed by exact volumetry after 3D-segmentation in 60 healthy individuals. Median values are indicated by the horizontal line. Box length shows interquartile range, whereas whiskers represent range of data. NS = non-significant.

Mean DRG volumes continuously increased from L3 to S1, whereas mean DRG volume of S2 was smaller than that of S1 (Figure 3). Paired *t*-test did not reveal any differences between right and left DRG volumes.

Normal values of DRG volumes were assessed by exact volumetry in all 60 participants (Table 1). Since we did not find any difference between right and left DRG, normal values are reported based on an analysis of both sides. DRG S2 was not included in the field of view (FOV) in four cases (right hand side) and five cases (left hand side). All other DRGs could be assessed. 

**Figure 4:**
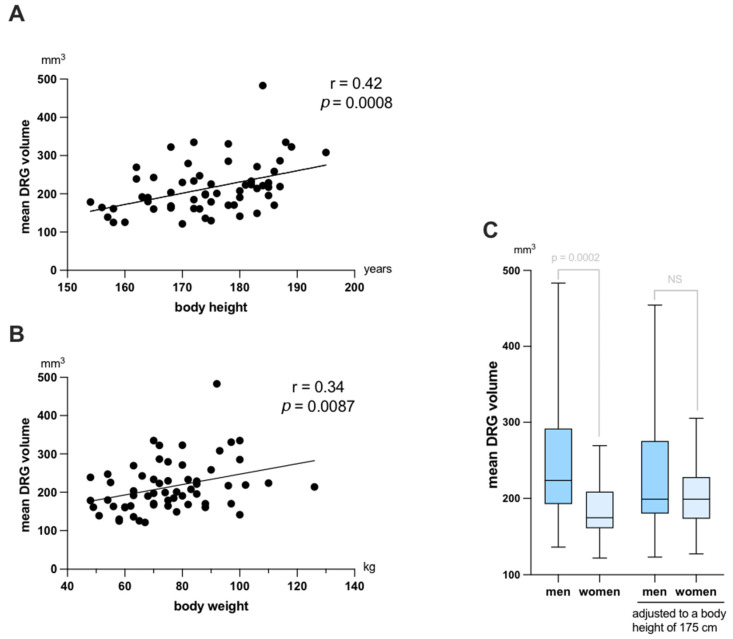
Correlation analyses of dorsal root ganglia (DRG) volume (mean of L3-S1) with the demographic variables (**A**) body height and (**B**) body weight. r = Pearson correlation coefficient. (**C**) Box-and-whisker plot of mean DRG volume in men and women as measured (left plots) and after correction to a body height of 175 cm (right plots) by linear regression. Median values are indicated by the horizontal line. Box length shows interquartile range, whereas whiskers represent range of data. NS = non-significant.

Correlation analyses revealed positive correlations of mean DRG volume (mean of L3 to S1, averaged over both sides) with both body height (r = 0.42; *p* = 0.0008) and body weight (r = 0.34; *p* = 0.0087) (Figure 4A,B). Associations of mean DRG volume with age (r = 0.21, *p* = 0.09) and body mass index (r = 0.18, *p* = 0.17) were not statistically significant. Mean DRG of men was significantly higher than that of women (244.4 vs. 183.4 mm^3^; *p* = 0.0002; Figure 4C). This could be attributed to men in our cohort being taller overall. No difference could be detected between men and women when corrected DRG values were compared, which were adjusted to a body height of 175 cm by linear regression (Figure 4C). 

### 3.3. Inter-Reader Reliability as Assessed for Each Method

In order to assess reliability, the volumes of the right and left DRG L3, L4, L5, S1 and S2 were measured in a subset of 25 randomly selected participants by two readers and using the three measurement methods exact volumetry, non-axis-corrected estimation and axis-corrected estimation. In two of these participants, both DRG S2 were outside the field of view (total = 1476 measurements).

Inter-reader reliability as assessed by interclass correlation coefficients was highest for exact volumetry (ICC = 0.99, 95% CI: 0.99–1.00), followed by axis-corrected estimation (ICC 0.98, 95% CI: 0.97–0.98) and non-axis-corrected estimation (ICC = 0.98, 95% CI: 0.97–0.98). According to Koo and Li, all values >0.9 may be considered to be in excellent agreement [21]. 

**Figure 5:**
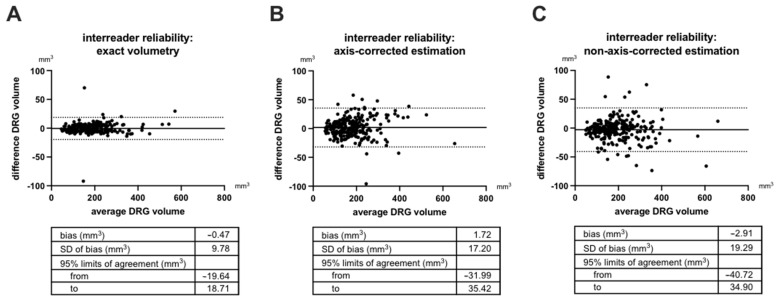
Bland-Altman plots of inter-reader reliability. Dorsal root ganglia (DRG) L3 to S2 were analyzed in 25 participants by two readers by exact volumetry (**A**), axis-corrected estimation (**B**), and non-axis-corrected estimation (**C**). SD = standard deviation.

In order to quantitatively assess the systematic and random error associated with different readers, we conducted a Bland–Altman analysis (Figure 5). This analysis also showed that inter-reader reliability is best for systematic volumetry, mainly due to a lower random error of exact volumetry (standard deviation of bias: 9.78 mm^3^) when compared with axis-corrected estimation and non-axis-corrected estimation (SD of bias: 17.20 mm^3^ and 19.29 mm^3^). Systematic error (bias) was also best for exact volumetry but practically negligible for all three methods (<3 mm^3^). Subsequently, the 95% limits of agreement were best for inter-reader reliability of exact volumetry (−19.64 mm^3^; 18.71 mm^3^) when compared to axis-corrected (−31.00 mm^3^; 35.42 mm^3^) and non-axis-corrected estimation (−40.72 mm^3^; 34.90 mm^3^).

### 3.4. Inter-Method Reliability

Inter-method reliability was assessed in the same subset of 25 participants for both readers separately. Since exact volumetry may be regarded as the gold-standard [13,14,18], we compared both methods of volume estimation (axis-corrected and non-axis-corrected) with exact volumetry. ICC-values for inter-method reliability are given in Table 2. According to Koo and Li [21], reliability of axis-corrected estimation may be considered as excellent (ICC: 0.91), based on the results of reader 1, or good (ICC: 0.88), based on reader 2, when compared with exact volumetry. Reliability of non-axis-corrected estimation may be considered as good, based on the results of both readers (ICC: 0.90 and 0.88).

**Figure 6:**
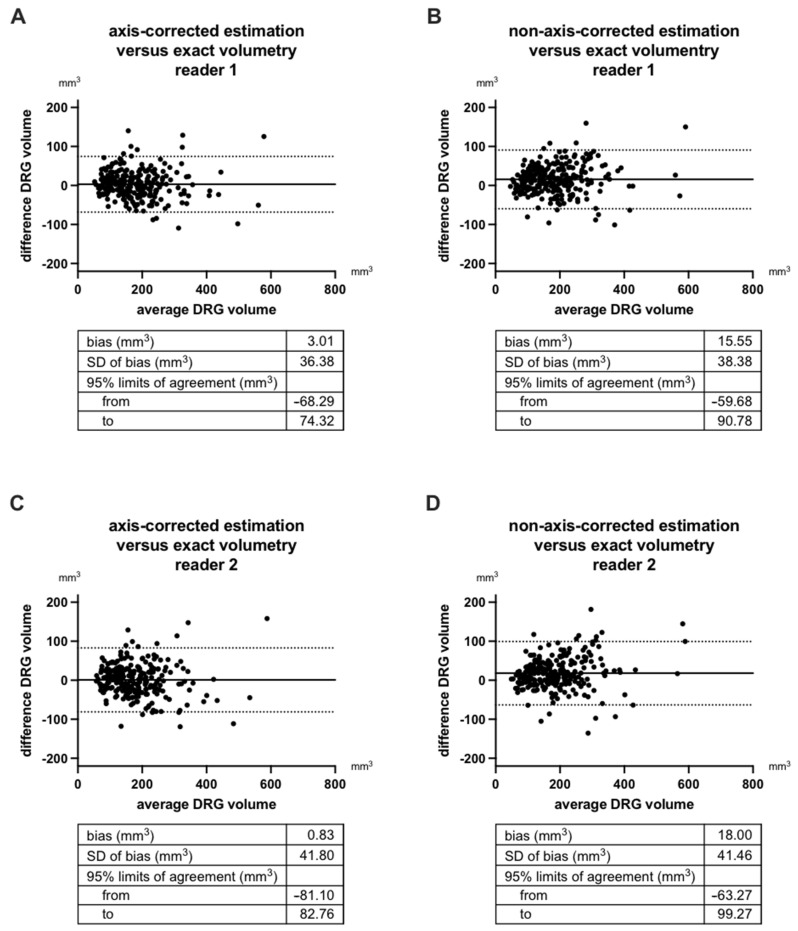
Bland–Altman plots of inter-method reliability comparing axis-corrected estimation with exact volumetry and non-axis corrected estimation with exact volumetry in reader 1 (**A**,**B**) and reader 2 (**C**,**D**). Positive values of difference DRG volume indicate overestimation, negative values indicate underestimation of DRG volume by the estimation formula when compared with exact volumetry. DRG = dorsal root ganglion. SD = standard deviation.

Bland–Altman plots of inter-method reliability are given in Figure 6. Systematic error (bias) of axis corrected estimation when compared with exact volumetry is practically neglectable in both readers (≤3 mm^3^; Figure 5A,C). Non-axis-corrected estimation, however, was associated with a systematic error (bias) of 15.55 mm^3^ in reader 1 and 18.00 mm^3^ in reader 2 when compared with the gold standard of exact volumetry. This means DRG volume is systematically overestimated by non-axis-corrected estimation. The random error (standard deviation of bias) of axis-corrected and non-axis-corrected estimation were comparable and ranged between 36.38 mm^3^ and 41.80 mm^3^. Subsequently, 95% limits of agreement ranged between −68.29 and 82.76 mm^3^ for axis-corrected estimation vs. exact volumetry and −63.27 and 99.27 mm^3^ for non-axis-corrected estimation vs. exact volumetry.

Since Bland–Altman analysis showed a systematic overestimation of DRG volume by non-axis-corrected estimation, we conducted an ANOVA to compare all measurement values by the three methods of volumetry for reader 1 and 2 separately (Supplementary Figure S2). For both readers, mean DRG volume obtained by non-axis-corrected estimation was larger compared to exact volumetry and also larger compared to axis-corrected estimation. No difference was observed between axis-corrected estimation and exact volumetry (Supplementary Figure S2).

As a supplementary analysis, DRG volume was additionally calculated based on a recently proposed formula and results were compared with exact volumetry [13]. In our cohort of 25 volunteers, the proposed formula systematically overestimated DRG volume by a bias of 127.70 mm^3^ (reader 1) and 125.00 mm^3^ (reader 2) when measurements were performed in axis-corrected reformations and 143.70 mm^3^ (reader 1) and 146.90 mm^3^ when measurements were performed in standard anatomical reconstructions.

## 4. Discussion

In this study, we provide normative values of lumbosacral DRG volumes based on a cohort of 60 healthy participants with correlations to demographic determinants and found associations of DRG volume with both body height and weight. Additionally, we quantify inter-method and inter-reader reliability of DRG volumetry by three different methods: exact volumetry based on 3D segmentation, axis-corrected estimation and non-axis corrected estimation. As a principal finding, non-axis-corrected estimation systematically overestimated DRG volume when compared with exact volumetry. In contrast, axis-corrected estimation was not associated with a relevant systematic error when compared with exact volumetry. Inter-reader reliability was high for all three methods but best for exact volumetry, which should be regarded the gold standard of DRG volume quantification.

DRG volume has become a key-marker in the quantification of DRG involvement, e.g., in Fabry disease, Oxaliplatin-induced PNP and Neurofibromatosis Type 2 [5,8,19,22,23]. In this study, we provide mean values of the lumbosacral DRG L3 to S2 as well as normal values based on the 3rd and 97th percentile of our cohort of 60 healthy volunteers. A potential application of our normal values may be illustrated when comparing them with the existing literature. Published mean DRG values in patients with neurofibromatosis type 2 (NF2) clearly exceed the 97th percentile of DRG volume in our cohort for the DRG L3, L4, L5 and S1, whereas a published a mean DRG value at S2 in patients with NF 2 is above the mean value in our healthy cohort but does not exceed our 97th percentile [8]. Additionally, published mean DRG values in patients with Fabry neuropathy exceed the 97th percentile of our cohort for the DRG L3, L4, and L5, while mean DRG volume at S1 and S2 in Fabry patients markedly exceed the mean values of our healthy participants but not the 97th percentile [5]. In a clinical setting, an unambiguous hypertrophy of the DRG beyond the here proposed normative values may therefore indicate a definite pathological finding such as NF2 or Fabry neuropathy [5,8]. However, published DRG volumes in patients with oxaliplatin-induced neuropathies do not exceed the here proposed normal values while still exceeding the values of the corresponding control group [19]. Besides differences in segmentation techniques and demographic determinants, which may contribute to differences in measurement values, “milder” hypertrophies may be missed when using cut-off values based on the 97th percentile on an individual patient basis. 

In this study, we assessed correlations of DRG volume with the demographic variables age, body height, weight, and body mass index (BMI). Of these four parameters, we found moderate positive associations for body height and weight with DRG volume. Similar associations have been described for the cross-sectional area of extremity nerves with body weight and height in studies using magnetic resonance neurography or ultrasound [11,24]. In children, a similar association of DRG volume with body height and weight has been described [25]. While age was also described to correlate with DRG volume in children, we did not find a similar association of age and DRG volume in adult participants [25]. In our cohort, DRG volume of men was significantly large than that of women. This may be explained by the association of DRG volume and body height, since men in our cohort were significantly larger than women. After calculating the correct DRG volume, which was adjusted to an arbitrary body height of 175 cm by linear regression, we did not observe any difference between DRG volume of men and women.

Another aim of this study was to assess both inter-reader reliability and inter-method reliability for three different methods of DRG volumetry. Bland–Altman analysis breaks down reliability into a systematic error (=bias), implying one reader or method systematically measures more than another and a random error (=standard deviation of bias) [26]. Assuming a normal distribution, both errors are used to calculate 95% limits of agreement, which allow us to predict to which extent measures by two readers or methods differ in 95% of cases. Interestingly, systematic error between readers was practically negligible (<3 mm^3^) for all three methods, implying the DRG contours could be unambiguously defined by the two readers. As expected, exact volumetry yielded the lowest random error and, subsequently, also the best 95% limits of agreement, which are almost half of those of non-axis corrected estimations and may serve as orientation values of inter-reader accuracy of DRG volumetry.

We analyzed inter-method reliability by comparing axis-corrected and non-axis-corrected volume estimation with the gold standard of exact volumetry. Notably, axis-corrected estimation was not associated with a relevant systematic error (bias) when compared with exact volumetry in Bland–Altman analysis. On the other hand, non-axis-corrected volume estimation systematically overestimated the DRG volume by on average 16 mm^3^ (reader 1) or 18 mm^3^ (reader 2). This bias may be considered an orientation value, when DRG volumes measured by non-axis-corrected estimation are interpreted and compared with exact volumetry or axis-corrected estimation. The random error of axis-corrected and non-axis corrected volume estimation was in a similar range. 

Our results regarding the systematic error of different types of DRG volumetry stand in contrast to a recent study by Weiner et al., that was published during the preparation of this manuscript [13]. In that study, accuracy of DRG volume estimation using different formulae was assessed in comparison with slice-by-slice volumetry. While the systematic error in our cohort for axis-corrected volume estimation was practically negligible, Weiner et al. reported an underestimation of DRG volume when using the standard formula (V = π/6 × A × B × C) for an ellipsoid in a cohort of 12 volunteers. While we both reported a high inter-reader reliability, a possible explanation for the different results might be the exact definition of the DRG borders by the respective readers. In light of their own results, Weiner et al. propose a novel correction formula for volume estimation of DRG (V = 2/3 × A × B × C + 75 mm^3^) that is derived from a cohort of 12 volunteers. While they also use this formula to calculate normal DRG values in a larger collective, a validation in comparison with exact volumetry in this larger cohort seems not to have been conducted. We therefore aimed to validate this formula in a supplementary analysis including 25 participants of our cohort and found that it led to DRG volume overestimation when compared with exact volumetry. This may also explain why Weiner et al. report higher normal values of DRG volume than we do. Our results, therefore, rather support continuing to use the conventional ellipsoid formula for DRG volume estimation instead of that novel correction formula. In line with Weiner et al. are our results regarding the association of body height and DRG volume and the finding of men having larger DRG than women. 

This study comes with methodical limitations. First, we only analyzed DRG volume in healthy participants. Since ICC values always depend on the variance of the parameter in the observed population, an analysis in patients with hypertrophic DRG may lead to different results. However, we would expect the absolute values of measurement accuracy as revealed by Bland–Altman analysis also in patients to be in similar ranges. Additionally, due to the dependence of the ICC on the variance of the parameter, ICC values calculated for each segment may differ from ICC values based on all analyzed segments [27]. This is another reason why we would like to focus on absolute measurement error as described by Bland–Altman analyses rather than on ICC values. Moreover, analyses were conducted by two well-trained readers. Analyses by other readers in a clinical setting may potentially lead to different values of reliability. Therefore, the here described values may rather serve as an orientation under ideal conditions. Furthermore, subjects in this study were examined once. An assessment of test retest-reliability using multiple scans of the same subjects would be desirable in future studies. Lastly, all quantitative MR parameters may be influenced by sequence parameters and the selected hardware. However, we expect the influence of hardware and sequence details for morphometric parameters to be rather small in comparison with other quantitative MR techniques such as diffusion tensor imaging or T2 relaxometry.

Future studies might examine not only the influence of demographic data to PNS morphology, but also other determinates such as physical activity or muscle mass and body fat percentage. Moreover, technical approaches that are capable to quantify DRG volumes throughout the spine by fully automatic segmentation and put it in relation to normative values would be highly desirable.

In conclusion, the here provided normative values of DRG volumes and the reported associations of DRG volume with body height and weight may provide a basis for future disease-specific studies. Moreover, we provide quantitative orientation values of measurement accuracy for three methods of DRG volumetry. While exact volumetry based on 3D segmentation performed the most precisely and should be regarded as the gold standard, axis-corrected volumetry was not associated with systematic over- or underestimation of the DRG volume. In contrast, non-axis-corrected volume estimation systematically overestimated the DRG volume. However, its reliability might still be considered sufficient, depending on the specific type of clinical application and the required degree of measurement accuracy.

## Figures and Tables

**Table 1:** Normal values of dorsal root ganglia (DRG) volume as assessed by exact volumetry after 3D-segmentation in 60 healthy individuals.

	L3	L4	L5	S1	S2
**Number of values (right + left)**	120	120	120	120	111
**Mean DRG volume (mm^3^)**	124.8	163.4	244.0	323.3	179.4
**Std. Deviation (mm^3^)**	51.1	58.5	76.9	124.5	107.1
**3% Percentile–97% Percentile (mm^3^)**	58.1–251.9	86.7–308.5	130.3–410.3	164.7–633.2	55.8–458.2
**Range (mm^3^)**	52.9–331.7	57.8–373.3	109.5–527.6	129.7–893.3	45.4–542.3

**Table 2:** Interclass-correlation coefficients (ICCs) for inter-method reliability.

	Reader 1	Reader 2
Axis-corrected estimation vs. exact volumetry	0.91 (0.89–0.93)	0.88 (0.85–0.91)
Non-axis-corrected estimation vs. exact volumetry	0.90 (0.84–0.93)	0.88 (0.81–0.92)

## Data Availability

The data that support the findings of this study are available on request from the corresponding author. The data are not publicly available due to restrictions, as they contain information that could compromise the privacy of the research participants.

## Supplementary Materials

The following supporting information can be downloaded at: https://www.mdpi.com/article/10.3390/diagnostics12071570/s1, Figure S1: Bland-Altman plots comparing DRG volume estimation by application of the formula proposed by Weiner et al., Figure S2: Comparison of DRG measurement values in 25 participants by two readers. Table S1: Demographic characteristics of study participants.

## Abbreviations

PNS (Peripheral nervous system), DRG (Dorsal root ganglion), MRN (MR-Neurography), PNP (Polyneuropathy), CNS (Central nervous system), SPACE (Sampling-Perfection-with-Application-optimized-Contrasts-using-different-flip-angle-Evolution), STIR (Short-Tau-Inversion-Recovery), DICOM (Digital Imaging and Communications in Medicine), FOV (Field of view), ICC (Interclass correlation coefficient), ANOVA (analysis of variance), BMI (Body mass index).
