# Supplementary material for: Dorsal Root Ganglia Volume—Normative Values, Correlation with Demographic Determinants and Reliability of Three Different Methods of Volumetry
Source: Diagnostics (Basel). 2022 Jun 28;12(7):1570. doi: 10.3390/diagnostics12071570 (PMC9323629; doi:10.3390/diagnostics12071570)
Supplement: Supplementary file 1 [file diagnostics-12-01570-s001.zip › diagnostics-1762602-supplementary.pdf]

Table S1: Demographic characteristics of study participants.

sex	age (years)	body height (cm)	body weight (kg)	body mass index (kg/m²)
female	23	175	55	18,0
male	25	185	85	24,8
male	25	195	93	24,5
male	25	187	102	29,2
female	25	157	51	20,7
female	25	168	56	19,8
male	26	174	63	20,8
female	26	172	60	20,3
female	26	164	54	20,1
male	28	183	78	23,3
female	30	168	70	24,8
male	31	180	100	30,9
female	31	158	49	19,6
male	33	189	80	22,4
female	33	154	48	20,2
male	35	188	100	28,3
male	36	186	97	28,0
male	37	185	85	24,8
female	38	180	80	24,7
female	38	160	65	25,4
male	40	172	70	23,7
female	41	168	82	29,1
female	46	158	58	23,2
female	47	164	68	25,3
male	48	183	126	37,6
male	48	182	110	33,2
female	48	173	54	18,0
female	48	179	70	21,8
male	49	171	75	25,6
male	50	178	100	31,6
male	50	187	72	20,6
female	50	156	75	30,8
female	51	174	70	23,1
male	54	183	80	23,9
female	54	165	60	22,0
female	54	173	88	29,4
male	58	176	78	25,2
female	58	163	63	23,7
male	59	185	96	28,0
male	59	178	88	27,8
male	62	182	82	24,8
male	62	184	85	25,1
female	62	172	70	23,7
male	65	168	72	25,5
female	65	168	62	22,0
female	66	170	75	26,0
male	67	178	97	30,6
male	68	186	90	26,0
female	69	162	63	24,0
female	69	174	74	24,4
female	70	162	48	18,3
female	71	165	66	24,2
male	72	172	77	26,0
male	73	181	72	22,0
male	73	180	83	25,6
female	73	175	58	18,9
female	75	170	67	23,2
male	77	175	75	24,5
male	79	184	92	27,2
female	79	168	63	22,3

A

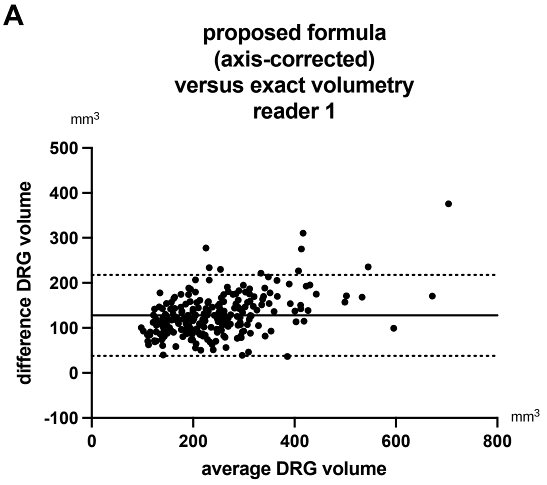

bias (mm^3)	127.70
SD of bias (mm^3)	45.87
95% limits of agreement (mm^3)	
from	37.79
to	217.60

B

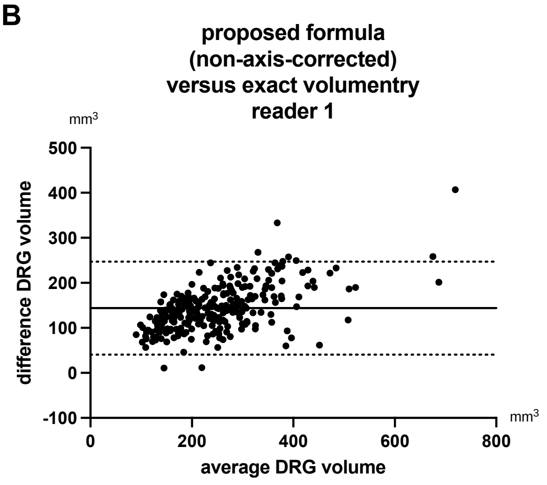

bias (mm^3)	143.70
SD of bias (mm^3)	52.69
95% limits of agreement (mm^3)	
from	40.39
to	246.90

C

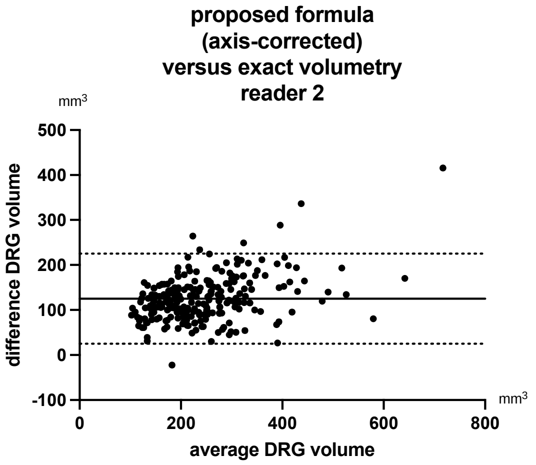

bias (mm^3)	125.00
SD of bias (mm^3)	51.06
95% limits of agreement (mm^3)	
from	24.97
to	225.10

D

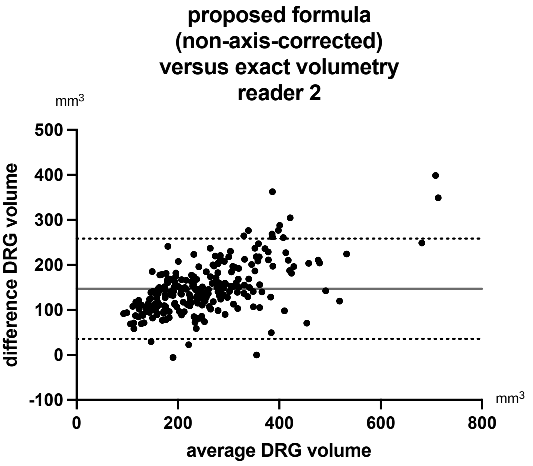

bias (mm^3)	146.90
SD of bias (mm^3)	56.84
95% limits of agreement (mm^3)	
from	35.50
to	258.30

Figure S1: Bland-Altman plots comparing dorsal root ganglion (DRG) volume estimation by application of the formula proposed by Weiner et al. [13] ($V = 2/3 \times A \times B \times C + 75\ mm^3$) with exact volumetry based on 3D segmentation in our cohort of 25 participants. The maximal diameters of the DRG in three spatial directions were measured in axis-corrected reformations (A and C) and standard anatomical reformations without axis-correction (B and D) for reader 1 and 2 respectively. Positive values for difference DRG volume indicate that the proposed formula overestimates DRG volume when compared with exact volumetry. SD = standard deviation.

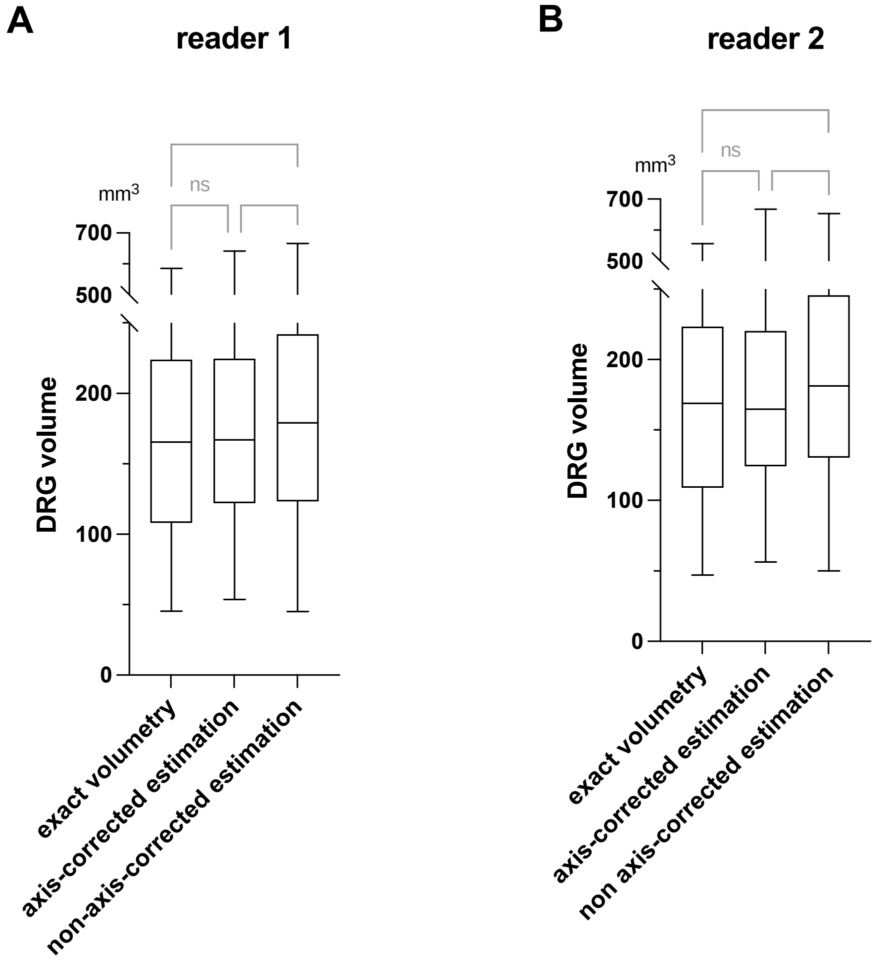

	reader 1			reader 2		
	exact volumetry	axis-corrected estimation	non-axis-corrected estimation	exact volumetry	axis-corrected estimation	non-axis-corrected estimation
number of values	246	246	246	246	246	246
mean (mm³)	178.8	181.8	194.4	179.3	180.1	197.3
standard deviation (mm³)	89.5	86.6	94.2	88.2	84.9	95.4

Figure S2: Comparison of DRG measurement values in 25 participants by reader 1 (A) and reader 2 (B). All measurement values as acquired by exact volumetry, axis-corrected estimation and non-axis-corrected estimation were compared by analysis of variance (ANOVA). **** $p < 0.0001$ after Tukey correction for multiple comparisons. ns = non-significant.
